# Preparation and Characterization of Double-Network Composite Hydrogels with Carboxymethyl Pachymaran in Promoting Wound Healing

**DOI:** 10.3390/foods15081285

**Published:** 2026-04-08

**Authors:** Haodong Wu, Xi Feng, Zhinan Mei, Wen Huang, Ying Liu

**Affiliations:** 1 College of Food Science and Technology, Huazhong Agricultural University, Wuhan 430070, China; wuhaodong1118@163.com; 2 Hubei Key Laboratory of Fruit& Quality Control, Huazhong Agricultural University, Wuhan 430070, China; 3 Department of Nutrition, Food Science, and Packaging, San Jose State University, San Jose, CA 95192, USA; xi.feng@sjsu.edu; 4 College of Plant Science and Technology, Huazhong Agricultural University, Wuhan 430070, China; meizhinan@mail.hzau.edu.cn

**Keywords:** *Poria cocos*, fungal polysaccharides, double-network hydrogel, bioactivity

## Abstract

Utilizing food-derived bioactive polysaccharides in advanced biomedical applications offers significant potential. To effectively harness the inherent bioactivity of *Poria cocos*, a renowned edible and medicinal fungus, we developed a multifunctional double-network composite hydrogel (CPS) via a feasible one-pot strategy. This was achieved by incorporating functional carboxymethyl pachymaran (CMP) into a matrix of food-grade sodium alginate (SA) and polyacrylamide (PAM). This formulation endows the hydrogel with excellent extensibility, rapid self-healing capabilities, and strong tissue adhesion, all while preserving the biological activity of the natural macromolecules. In a mouse full-thickness skin defect model, the CPS significantly accelerated wound recovery, achieving a healing rate of 51.17 ± 4.87% by day 7. Mechanistically, the food-derived CMP synergistically promoted skin tissue regeneration by downregulating the expression of the early pro-inflammatory cytokine TNF-α and upregulating the angiogenic marker CD31, thereby actively modulating the local microenvironment. Ultimately, these findings demonstrate the viability of using edible fungal polysaccharides as primary bioactive components in advanced wound dressings, providing a novel approach for utilizing food macromolecules in biomedicine.

## 1. Introduction

In recent years, the high-value utilization of food-derived bioactive macromolecules, particularly natural polysaccharides, in biomedical applications has attracted widespread attention [1,2]. Traditional wound dressing materials like gauze and bandages, while widely used, can cause wound dryness, pain, and delayed re-epithelialization [3,4,5]. Moreover, they offer insufficient exudate absorption and wound protection, and their poor flexibility is not suitable for irregular wound surfaces [6,7]. Consequently, there is an urgent need to develop innovative multifunctional dressings that not only preserve a moist healing environment and permit gas exchange but also actively integrate advanced functionalities, such as robust antioxidant and antimicrobial capabilities, to effectively facilitate tissue regeneration [8,9,10]. Hydrogels, with their high water content, biocompatibility, and structural similarity to bodily macromolecules, are perfect candidates, facilitating active ingredients diffusion and creating a moist healing environment [11,12,13].

*Poria cocos* is a renowned edible and medicinal macro-fungus with a long history of consumption as a functional food and traditional medicine. Its primary bioactive component, *P. cocos* polysaccharide (PCP), exhibits potent traditional pharmacological properties, including antioxidant, anti-inflammatory, and immunomodulatory effects [14,15]. These inherent biological activities make PCP highly promising for addressing the severe oxidative stress and prolonged inflammation typical of complex wound microenvironments [16,17]. However, the poor water solubility of natural PCP significantly limits its application in targeted delivery systems such as hydrogels [18]. Carboxymethylation modification effectively overcomes this limitation, increasing the water solubility of Poria polysaccharides (>80%) while retaining and even enhancing their bioactivity and functional group diversity [19].

To effectively harness and sustain the inherent bioactivity of modified carboxymethyl pachymaran (CMP) in vivo, an optimal delivery matrix is required. Single-component hydrogels often lack sufficient mechanical strength and tissue adhesion [20,21]. To address this, double-network (DN) hydrogels incorporating food-grade natural polymers have emerged as a superior strategy [22,23]. While conventional PAM/SA hydrogels typically incorporate polysaccharides as passive structural fillers, the present CPS system employs carboxymethyl pachymaran (CMP) as its core bioactive agent. In contrast to traditional drug-delivery platforms that rely on the burst release of exogenous synthetic agents, harnessing the inherent bioactivity of this edible natural macromolecule provides a safer and sustainable strategy for modulating biological responses to achieve long-term therapeutic efficacy [24,25]. Integrating structural excellence with the functional benefits of food-derived macromolecules offers a highly promising strategy for advanced wound management. Therefore, the objective of this study was to develop a novel multifunctional double-network hydrogel dressing (designated as CPS) by incorporating bioactive CMP into a polyacrylamide (PAM) and sodium alginate (SA). In this system, the PAM/SA backbone provides a stable network for rigidity and tissue adhesion, while the CMP acts not only as the core bioactive agent for pro-healing but also provides numerous dissipation sites through long-chain entanglement and reversible hydrogen/carboxyl interactions, thereby significantly enhancing the hydrogel’s toughness. This study comprehensively assessed the physicochemical properties of the CPS and its potential to accelerate wound healing through in vitro and in vivo models, providing new insights into the biomedical valorization of edible fungal polysaccharides.

## 2. Materials and Methods

CMP (β-(1 → 3)-D-glucan, purity ≥85%, carboxymethyl substitution degree of 0.69, molecular weight about 780,000 Da) was purchased from Wuhan Runge Bio Co., Ltd. (Wuhan, China), and the structural information was provided by the supplier (Figures S4 and S5 in the Supplementary Materials). Acrylamide (AM), N,N′-Methylenebisacrylamide (BIS), Sodium Alginate (SA) from Aladdin (Shanghai, China), and N,N,N′,N′-Tetramethylethylenediamine (TEMED) were obtained from Aladdin (Shanghai, China). Potassium persulfate (KPS) was sourced from Luoen (Shanghai, China). All other chemical solvents and reagents were purchased from commercial sources.

### 2.1. Hydrogel Preparation

Sodium alginate (SA) and carboxymethyl pachymaran (CMP) were dissolved in 100.0 mL of deionized water to maintain a constant total polysaccharide concentration of 1.0 wt% (*w*/*v*). Specifically, the weight percentages of SA and CMP were adjusted to 0.83 wt%:0.17 wt%, 0.75 wt%:0.25 wt%, and 0.50 wt%:0.50 wt% (*w*/*v*) to prepare the precursor solutions for CPS1, CPS2, and CPS3, respectively. The mixtures were stirred at 50–60 °C for 4 h until complete dissolution and then degassed for 24 h. Subsequently, acrylamide (8.00 g) was added and stirred at 60 °C until homogeneous. To initiate polymerization, 100 mg of BIS and 100 mg of KPS (each pre-dissolved in 10.0 mL of water) were introduced, followed by the dropwise addition of 100 μL of TEMED. The resulting solutions were poured into molds and cured at room temperature for approximately 4 h to obtain the CPS double-network hydrogels.

### 2.2. Characterization of Hydrogels

The chemical structures and intermolecular interactions of the hydrogels were analyzed using a Fourier Transform Infrared (FT-IR) spectrometer (IS50, Suzhou Opus Plasma Technology Co., Ltd., Suzhou, China). Briefly, 2 mg of freeze-dried hydrogel sample was mixed with KBr (dried overnight at 49 °C) at a mass ratio of 1:100, ground into fine powder, and pressed into a pellet. The spectra were recorded in the wavenumber range of 4000 to 400 cm^−1^ at a resolution of 4 cm^−1^ with 32 cumulative scans per sample, utilizing air scanning as the background.

To observe the internal cross-sectional morphology, the hydrogel samples were pre-frozen at −80 °C for 2 h and subsequently lyophilized in a vacuum freeze-dryer for 48 h to preserve their native porous architectures. The freeze-dried samples were then fractured, and their cross-sectional surfaces were sputter-coated with gold for 45 s at a current of 20 mA. The microscopic structures were examined using a scanning electron microscope (SEM, Quanta-200, FEI, Eindhoven, The Netherlands) operating at an accelerating voltage of 10 kV, an emission current of 10 mA, and a working distance of 25 mm.

### 2.3. Hydrogel Adhesion Experiments

Hydrogel adhesion experiments were performed using porcine skin pre-washed with PBS, and the adhesive performance was evaluated via lap-shear testing. Hydrogel samples (20 mm × 20 mm) were sandwiched between two pieces of porcine skin and gently pressed for 5 s to ensure uniform contact. The assembled specimens were then incubated in a humid environment for 1 h prior to testing to maintain consistent adhesion conditions.

### 2.4. Swelling Behavior of Hydrogels

The freeze-dried sample (20 ± 1 mg) was soaked in DIW at 37 °C to swelling equilibrium. The mass before and after swelling was measured, and the swelling ratio was calculated accordingly.

### 2.5. Cytotoxicity of L929 Cells 

The in vitro cytotoxicity of the hydrogels was evaluated using an MTT assay with L929 fibroblasts. Briefly, cells were seeded in 96-well plates (1 × 10^4^ cells/well) and equilibrated in complete medium (MEM-*α* supplemented with 10% FBS) at 37 °C and 5% CO_2_ for 24 h. To prepare the leachables, sterilized hydrogel samples (500 μL) were incubated in 5 mL of the culture medium at 37 °C for 24 h to obtain the conditioned extract, which was further sterilized under UV irradiation for 2 h. Subsequently, the initial culture medium was aspirated and replaced with 100 μL of the hydrogel extract, followed by an additional 24 h incubation. To quantify cell viability, 10 μL of MTT reagent was added to each well and incubated for 4 h. The supernatant was then carefully removed, and 100 μL of dimethyl sulfoxide (DMSO) was introduced to solubilize the formazan crystals. After 5 min of agitation, the absorbance at 490 nm was recorded using a microplate reader.

### 2.6. In Vivo Wound Healing Experiment of CPS

Male Kunming (KM) mice (6-week-old) were obtained from the Animal Center of Huazhong Agricultural University and housed in an SPF-grade facility with ad libitum access to standard chow and water. After a one-week acclimation period, the mice were randomly assigned to five experimental groups (*n* = 10 per group): (i) Sham group (uninjured), (ii) Negative control (untreated model), (iii) Positive control (EGF-loaded hydrogel), and (iv) CPS treatment groups. Under anesthesia, a circular full-thickness skin defect (diameter: 6 mm) was surgically induced on the depilated dorsal region of each mouse. During the healing period, wound sites were cleaned daily with sterile saline, and dressings were replaced to maintain a consistent microenvironment. Tissue samples were harvested at predetermined intervals (days 3, 6, and 9 post-surgery). One portion of the harvested tissue was immediately cryopreserved at −80 °C for total protein (TP) and hydroxyproline (Hyp) quantification, while the remaining specimens were fixed in 4% (*w*/*v*) paraformaldehyde for histopathological and immunohistochemical (IHC) evaluations. All procedures were conducted in strict accordance with the ARRIVE guidelines and Directive 2010/63/EU, and were approved by the Ethics Committee of Huazhong Agricultural University (No. HZAUMO-2025-0383).

### 2.7. Statistical Analysis

Statistical analysis was conducted using SPSS 17.0 and GraphPad Prism 8.0.2 software. All quantitative experimental results are expressed as the mean ± standard deviation (SD). Statistical significance among multiple groups was determined using a one-way analysis of variance (ANOVA) followed by Tukey’s post hoc test for multiple comparisons. A *p*-value of less than 0.05 was considered statistically significant (* *p* < 0.05, ** *p* < 0.01, *** *p* < 0.001, and **** *p* < 0.0001).

## 3. Results

### 3.1. The Microstructure and Mechanical Robustness of CPSs

**Figure 1:**
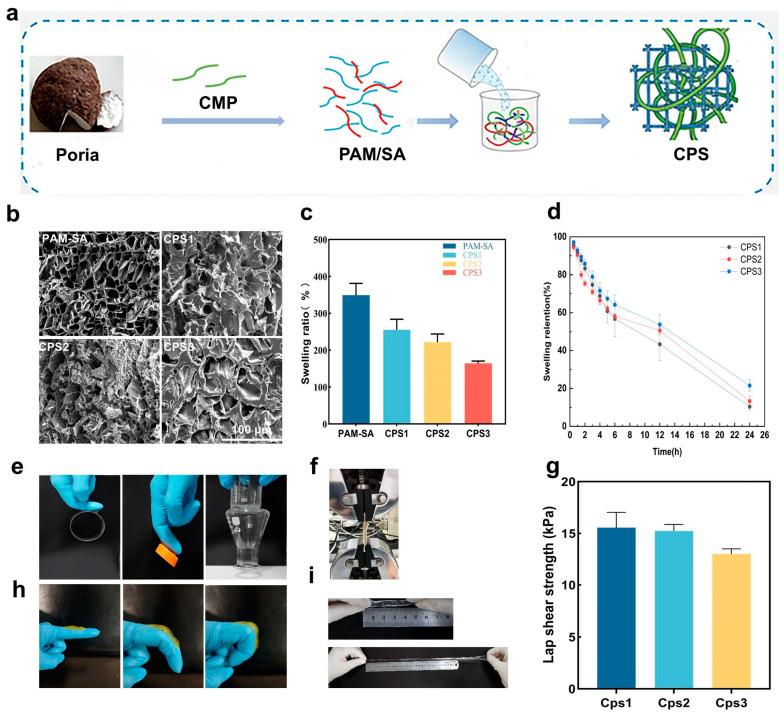
Fabrication and characterization of CPS: (**a**) The schematic diagram of preparation of the CPS. (**b**) SEM images of hydrogels. (**c**) Hydrogel swelling. (**d**) Hydrogel drying. (**e**) Hydrogel adhesion test. (**f**,**g**) Lap shear test. (**h**) Finger-bending test. (**i**) Hydrogel tensile test. Data are presented as mean ± SD (*n* = 5).

A series of CPSs (CPS1, CPS2, and CPS3) was fabricated via a one-pot polymerization process by incorporating varying mass fractions of CMP with sodium alginate (SA) and acrylamide (AM) (Figure 1a). The internal microstructure of a hydrogel dressing dictates its capacity for exudate absorption and nutrient diffusion, both vital for maintaining a healthy wound microenvironment [26]. SEM analysis was performed, as seen in Figure 1. Figure 1b revealed that the incorporation of CMP transformed the regular PAM-SA matrix into a dense, randomly distributed 3D porous network. Specifically, the high-molecular-weight CMP chains act as molecular struts within the PAM/SA framework, fostering a robust interpenetrating polymer network (IPN) that effectively anchors the dynamic constituents. Smaller pore sizes decrease water evaporation to maintain moisture, interconnected porous architectures enhance the diffusion of air, nutrients, and macromolecules, supporting the proliferation and communication of functional cells. Forming such polysaccharide-based IPN structures enhances the overall mechanical stability of the hydrogel through abundant non-covalent interactions [27]. This structural refinement was corroborated by the equilibrium swelling tests (Figure 1c,d), where the swelling ratio negatively correlated with CMP content. The swelling ratio negatively correlated with the CMP content; higher CMP proportions led to a more compact network, thereby moderately reducing the swelling capacity. This indicates that an appropriate ratio of SA and CMP forms a dense, ordered three-dimensional network structure that balances structural integrity with sufficient fluid uptake. The tissue adhesion of hydrogels is crucial for their application as wound dressings [28,29]. Benefiting from the synergistic hydrogen bonding and electrostatic interactions mediated by carboxyl and hydroxyl groups, the CPSs exhibited versatile adhesion to diverse substrates and biological tissues (Figure 1e), achieving a lap shear strength of 15 kPa (Figure 1f,g). Furthermore, the hydrogels maintained structural integrity under six-fold stretching (Figure 1i) and dynamic finger bending 60–180° (Figure 1h). This flexibility ensures stable wound coverage at high-strain joint sites while minimizing secondary trauma during dressing removal.

### 3.2. Physical Properties of Hydrogels

**Figure 2:**
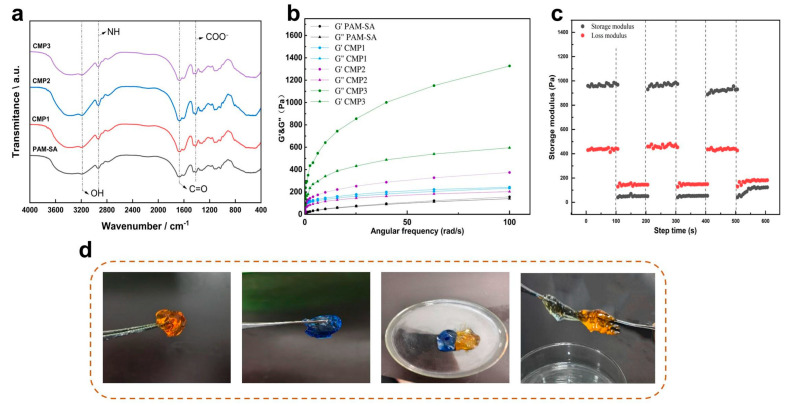
Rheological characterization and self-healing of hydrogels: (**a**) The FT-IR spectra of hydrogels. (**b**) Hydrogel storage and loss modulus. (**c**) Hydrogel cyclic loading. (**d**) Hydrogel self-healing. Data are presented as mean ± SD (*n* = 5).

FT-IR spectroscopy (Figure 2a) revealed complex intermolecular interactions within the CPS networks. The characteristic broad absorption at 3200–3600 cm^−1^ (-OH and -NH stretching) exhibited a blue shift and increased intensity with rising CMP content, indicating enhanced hydrogen-bonding intensity between the functional groups of CMP and the PAM/SA matrix. The amide I band (*C=O* stretching) of PAM near 1640 cm^−1^ also showed subtle shifts, suggesting synergistic hydrogen bonding and electrostatic interactions between CMP carboxyl groups and PAM amides [30,31]. Furthermore, the intensified *COO^−^* antisymmetric stretching vibrations in the 1400–1600 cm^−1^ range reflected the enriched carboxyl content, which likely facilitates ionic coordination and secondary crosslinking. These multi-level non-covalent interactions, coupled with CMP-induced chain entanglement, construct a robust sacrificial bond system that enhances energy dissipation and structural stability within the double-network framework.

Rheological analysis (Figure 2b) confirmed the elastic-dominated behavior (*G′* > *G″*) of all CPSs [32]. Notably, the storage modulus (*G’*) was significantly positively correlated with CMP content; the CPS3 sample reached 1300 Pa at 100 rad/s, a 13-fold enhancement over the PAM-SA control of 100 Pa. This enhanced network rigidity and energy storage capacity are attributed to the dense hydrogen-bonding crosslinks and topological entanglements mediated by the abundant hydroxyl and carboxyl groups on the CMP chains. The self-healing efficiency was evaluated via cyclic high-low strain testing (Figure 2c). Upon shifting from 1000% to 1% strain, both *G’* and *G″* recovered to their initial levels within 100 s, maintaining stability over multiple cycles. This self-healing mechanism originates from the rapid regeneration of dynamic non-covalent crosslinks at fracture interfaces. During this process, CMP chains provide essential structural support, preventing the hydrogel network from collapsing under high-strain dissociation (Figure 2d). Unlike to conventional dynamic-bond systems, this CMP-reinforced network achieves high-efficiency recovery without external stimuli, demonstrating significant toughness and long-term service potential in dynamic biomechanical environments.

### 3.3. In Vitro Biocompatibility of CPS

**Figure 3:**
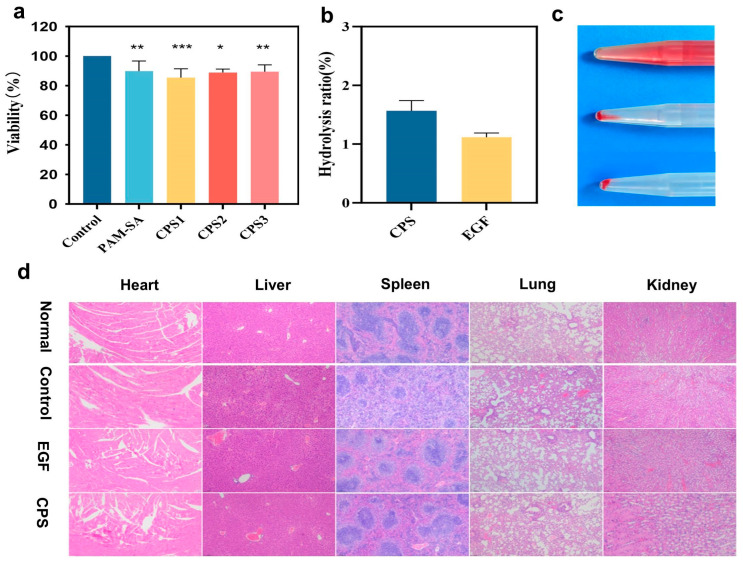
Safety assessment of hydrogels: (**a**) Cell viability. (**b**,**c**) Blood compatibility. (**d**) Effects on the heart, liver, spleen, lung, and kidney of mice. EGF is a commercially available gel containing epidermal growth factor. Data are presented as mean ± SD (*n* = 3). (* *p* < 0.05, ** *p* < 0.01 and *** *p* < 0.001).

A fundamental prerequisite for the application of food-derived biomacromolecules in tissue engineering is their excellent cytocompatibility, which is essential for facilitating effective tissue repair [33]. The biosafety of the CPSs was rigorously evaluated through cytotoxicity, hemocompatibility, and systemic toxicity assays. MTT assays (Figure 3a) demonstrated that L929 fibroblasts maintained high viability (84.5%) after 72 h of incubation with CPS extracts, confirming the negligible cytotoxicity of the hydrogel components. CMP and SA are naturally occurring edible carbohydrates. Their physiological degradation byproducts are primarily non-toxic oligosaccharides or monosaccharides. These can be easily metabolized or excreted through normal physiological pathways, highlighting the dressing’s strong potential for clinical translation. The hemolysis rate (Figure 3b,c) was significantly below the 5% threshold, with the CPS group exhibiting a pale-yellow supernatant similar to the saline control, indicating excellent hemocompatibility. This hemocompatibility is largely due to the gentle physical interactions within the polysaccharide network and the abundant negatively charged carboxyl groups from both SA and CMP, which are known to exert a repulsive force against the negatively charged surfaces of erythrocytes, thereby preserving red blood cell membrane integrity [34]. Finally, H&E staining of major organs (heart, liver, spleen, lung, and kidney) harvested on postoperative day 9 (Figure 3d) revealed no discernible inflammatory infiltration, cellular aggregation, or pathological alterations. These results collectively demonstrate that CPSs and their degradation byproducts possess superior biocompatibility and systemic safety for clinical wound management.

### 3.4. In Vivo Wound Healing Experiment of CPS

**Figure 4:**
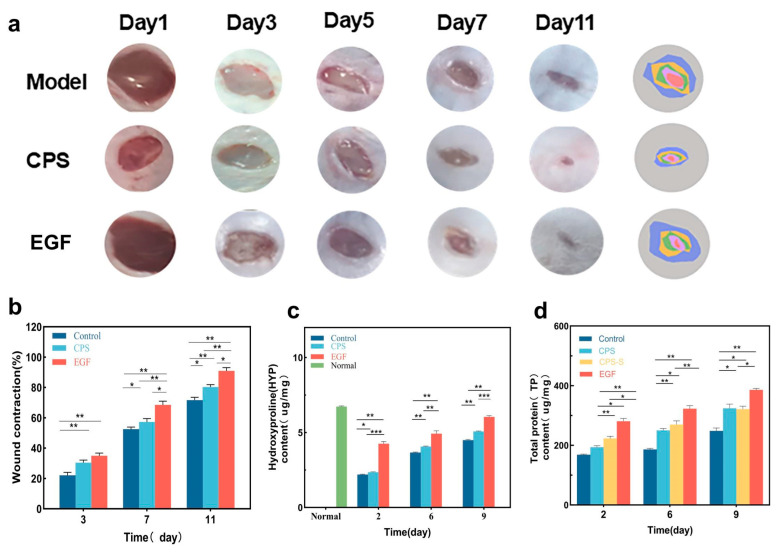
Wound healing outcomes under different treatments: (**a**) Representative images of mouse wound healing. (**b**) Quantitative analysis of mouse wound healing. (**c**) Hydroxyproline (HYP) content in mouse wound skin. (**d**) Total protein (TP) content in mouse wound skin. EGF is a commercially available gel containing epidermal growth factor. *** *p* < 0.001, ** *p* < 0.01, * *p* < 0.05. Data are presented as mean ± SD (*n* = 3).

The primary objective of advanced bioactive dressings is to accelerate the physiological phases of tissue repair in vivo. The therapeutic efficacy of the CPSs was evaluated using a mouse full-thickness skin defect model (Figure 4a). All treatment groups demonstrated accelerated wound contraction compared to the blank control. Notably, the CPS group achieved a wound closure rate of 51.18 ± 4.86% by day 7, showing regenerative capacity comparable to the EGF-positive control (Figure 4b). By day 11, while the control group still exhibited open wounds, the CPS and EGF groups reached near-complete healing (92.28 ± 5.01% for EGF). SA/PAM provides an optimal moist and breathable environment that prevents scab-induced delayed epithelialization, while the incorporated food-derived CMP serves as an active biological modulator, prompting the early transition from the inflammatory phase to the proliferative phase. This macroscopic recovery was supported by the quantitative analysis of hydroxyproline (Hyp) and total protein (TP) content, which are key indicators of collagen deposition and granulation tissue formation [35]. As shown in (Figure 4c,d), both Hyp and TP levels in the CPS group increased progressively and were significantly higher than those in the blank control from day 2 to day 9 (*p* < 0.05). The robust elevation of Hyp suggests that the bioactive CMP within the hydrogel matrix potentially stimulates fibroblast activity, accelerating the conversion of proline to hydroxyproline for stable collagen triple-helix formation. The synchronized elevation of Hyp and TP suggests that the CPS promotes the synthesis and accumulation of extracellular matrix (ECM) components, providing the necessary structural foundation for rapid skin tissue regeneration.

### 3.5. Immunofluorescence and Wound Staining

**Figure 5:**
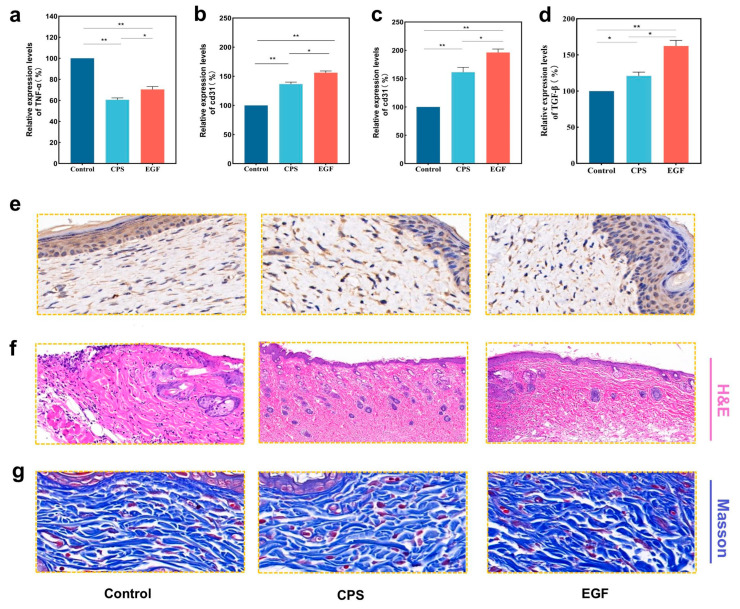
Fluorescence analysis and staining of wound skin: (**a**) TNF-α fluorescence analysis. (**b**) CD-31 fluorescence analysis on day 3. (**c**) CD-31 fluorescence analysis on day 9. (**d**,**e**) TGF-β immunohistochemical analysis. (**f**) Hematoxylin and eosin staining on day 6. (**g**) Masson’s trichrome staining on day 6. ** *p* < 0.01, * *p* < 0.05. Data are presented as mean ± SD (*n* = 3).

The timely transition from the inflammatory to the proliferative phase is a critical bottleneck in wound repair. Prolonged inflammation often leads to excessive reactive oxygen species and delayed healing [36]. The molecular mechanisms underlying accelerated wound repair were further elucidated via *TNF-α* (pro-inflammatory) and CD31 (angiogenic) markers. On day 3, *TNF-α* expression in the CPS group was significantly downregulated to 60.56% of the control level (Figure 5a and Figure S1), consistent with the alleviated inflammatory infiltration observed in H&E staining. Based on this immunofluorescence data and the existing literature, we hypothesize that this anti-inflammatory effect is associated with the recognition of CMP by Dectin-1 receptors on macrophages. This interaction likely facilitates an M1-to-M2 phenotypic switch, subsequently modulating the NF-κB signaling pathway [37]. The formation of dense microvascular networks is essential for delivering oxygen and nutrients to the nutrient-deprived wound bed. Immunofluorescence staining of *CD31*, a specific endothelial cell marker, revealed a marked increase in angiogenic activity in the CPS group (161.44% relative intensity) compared to the blank control on day 3 (Figure 5b,c, Figure S2 and Figure S3). We postulate that this correlative acceleration in angiogenesis may be structurally linked to the biomimetic features of the food-grade hydrogel matrix. Through electrostatic interactions, this matrix may potentially sequester and stabilize endogenous cationic growth factors secreted by local cells [38,39]. This effect enhances localized angiogenesis and ensures a continuous supply of oxygen and nutrients, thereby synergistically promoting tissue regeneration [40,41].

The regenerative quality of the healed tissue was further evaluated using TGF-β expression and histopathological analysis. Immunohistochemical staining (Figure 5d,e) revealed that CPS treatment significantly increased TGF-β expression by approximately 20% compared to the blank control (*p* < 0.05). This elevation in TGF-β levels is instrumental in driving fibroblast proliferation and enhancing granulation tissue formation [42,43]. H&E staining on day 6 (Figure 5f) corroborated these findings; the CPS group exhibited faster re-epithelialization, more organized connective tissue, and a higher density of regenerated skin appendages, such as mature hair follicles. The emergence of these appendages indicates not only morphological defect closure but also the functional restoration of the skin’s physiological microenvironment. Furthermore, Masson’s trichrome staining (Figure 5g) illustrated the profound impact of the CPSs on collagen remodeling. While the control group showed loose and disordered collagen fibers, the CPS group displayed densely packed, well-aligned collagen bundles. This accelerated structural maturation highlights the dual functionality of the CPS dressing: its porous, high-water-retention architecture provides a biomimetic moist physical barrier for exudate management, while the released food-derived CMP actively participates in local signaling, synergistically fostering rapid, high-quality skin regeneration.

## 4. Discussion

To provide a broader context, the performance of the CPS was benchmarked against recent state-of-the-art wound dressings. In terms of tissue adhesion, while specialized polydopamine (PDA)-integrated networks or dynamic metal-coordinated dextran/gelatin systems can achieve superior lap-shear strengths of approximately 20–27 kPa [44], the CPS exhibits a moderate and clinically effective adhesion (~15 kPa). This outperforms certain advanced smart hydrogels (~10 kPa) and ensures reliable conformability to wet wound beds while avoiding secondary mechanical trauma during dressing changes [45]. Regarding therapeutic efficacy, many contemporary advanced hydrogels achieve remarkable wound closure rates (83–98.2%) primarily by functioning as delivery vehicles for exogenous therapeutic payloads, such as specific drugs, amino acids (e.g., glycine), or metal–organic frameworks (MOFs) [46]. Strikingly, the CPS demonstrates a highly competitive healing trajectory (51.18 ± 4.86% closure at Day 7 and near-complete healing by Day 11) relying exclusively on the intrinsic immunomodulatory bioactivity of the food-derived CMP polysaccharide. This inherent bioactivity elegantly eliminates the need for complex drug-loading strategies, thereby minimizing potential long-term systemic toxicity and significantly facilitating its potential for clinical translation. Consequently, we did not use PAM/SA gels without added CMP as a control group in our experiment; however, as our understanding of the biological effects remains limited, this aspect requires further refinement in future work.

However, it is important to note that our immunofluorescence data regarding TNF-α and CD31 represent strong correlative observations rather than definitively proven causal pathways. While these outcomes align with theoretical models of macrophage polarization and growth factor sequestration, the current study focuses primarily on material characterization and the macroscopic evaluation of therapeutic efficacy. The precise molecular signaling pathways and direct protein–polysaccharide interactions remain theoretical; therefore, validating these mechanisms through specific gene knockout models or targeted receptor-blocking assays constitutes a critical focus for our future research.

Furthermore, the sustained in vivo therapeutic efficacy and bioactivity retention of the hydrogel are inherently linked to its degradation profile. Because CMP is integrated into the PAM/SA matrix primarily via physical entanglements and non-covalent interactions rather than permanent covalent bonds, the bioactive CMP is gradually released as the hydrogel network undergoes physiological swelling and erosion. This reliance on mild physical dissociation without chemical cleavage effectively preserves the structural integrity and inherent immunomodulatory bioactivity of the food-derived CMP upon release. Although the physiological degradation byproducts of these macromolecules are primarily non-toxic oligosaccharides, the lack of exact in vitro CMP release kinetics represents a limitation of the present study. Future investigations will prioritize tracking the specific pharmacokinetic release profile, in vivo metabolic fate, and degradation kinetics of CMP to comprehensively assess its long-term biosafety and clinical translation potential.

Finally, while the standard healthy murine full-thickness defect model used in this study successfully established the baseline biocompatibility, structural stability, and fundamental regenerative capacity of the CPS, we acknowledge limitations regarding its immediate clinical translation. Chronic wounds, such as diabetic ulcers or severe burns, feature highly complex microenvironments characterized by prolonged inflammation and impaired angiogenesis. Given the robust immunomodulatory and pro-angiogenic potential of the CMP-integrated matrix observed herein, the CPS holds significant promise for mitigating the harsh conditions of chronic wounds. However, translating these baseline findings requires rigorous validation in such diseased models. Advancing this food-grade biomaterial into clinically relevant chronic wound models to systematically evaluate its performance under severe oxidative and inflammatory stress will be a primary objective for our ongoing investigations.

## 5. Conclusions

In summary, this study successfully developed a novel bioactive wound dressing by utilizing *Poria cocos* polysaccharides, a functional food ingredient, as the core active component. Fabricated via a facile one-pot strategy, the resulting CPS double-network hydrogel (incorporating CMP, sodium alginate, and polyacrylamide) exhibits exceptional tissue adhesion, high stretchability, and rapid self-healing capabilities. In vivo evaluations on a murine full-thickness skin defect model unequivocally demonstrated that the CPS accelerates wound closure, enhances ordered collagen deposition, and restores skin appendages more effectively than traditional blank treatments. The therapeutic efficacy is driven by the synergistic ability of the hydrogel to provide a moist physical barrier while the released CMP biologically orchestrates early anti-inflammatory responses and promotes localized angiogenesis. Ultimately, this work provides a feasible and highly promising approach for fabricating advanced biomaterials, highlighting the immense potential of integrating the structural integrity of food-grade polymers with the inherent bioactivity of edible natural polysaccharides.

## Data Availability

The original contributions presented in this study are included in the article/Supplementary Materials. Further inquiries can be directed to the corresponding authors.

## Supplementary Materials

The following supporting information can be downloaded at: https://www.mdpi.com/article/10.3390/foods15081285/s1, Figure S1. TNF-α fluorescence analysis. Figure S2. CD-31 fluorescence analysis on day 3. Figure S3. CD-31 fluorescence analysis on day 9. Figure S4. NMR of CMP. Figure S5. The structural formula of CMP.

## Abbreviations

The following abbreviations are used in this manuscript:

CMP	Carboxymethyl pachymaran
CPS	CMP/PAM/SA
AM	Acrylamide
SA	Sodium alginate
TEMED	N,N,N′,N′-Tetramethylethylenediamine
BIS	N,N′-Methylenebisacrylamide
KPS	Potassium persulfate
TP	Total protein
HYP	Hydroxyproline
