# Supplementary material for: Preparation and Characterization of Double-Network Composite Hydrogels with Carboxymethyl Pachymaran in Promoting Wound Healing
Source: Foods. 2026 Apr 8;15(8):1285. doi: 10.3390/foods15081285 (PMC13115381; doi:10.3390/foods15081285)
Supplement: Supplementary file 1 [file foods-15-01285-s001.zip › foods-4217099-supplementary.pdf]

Supporting Information

In Vivo fluorescence analysis

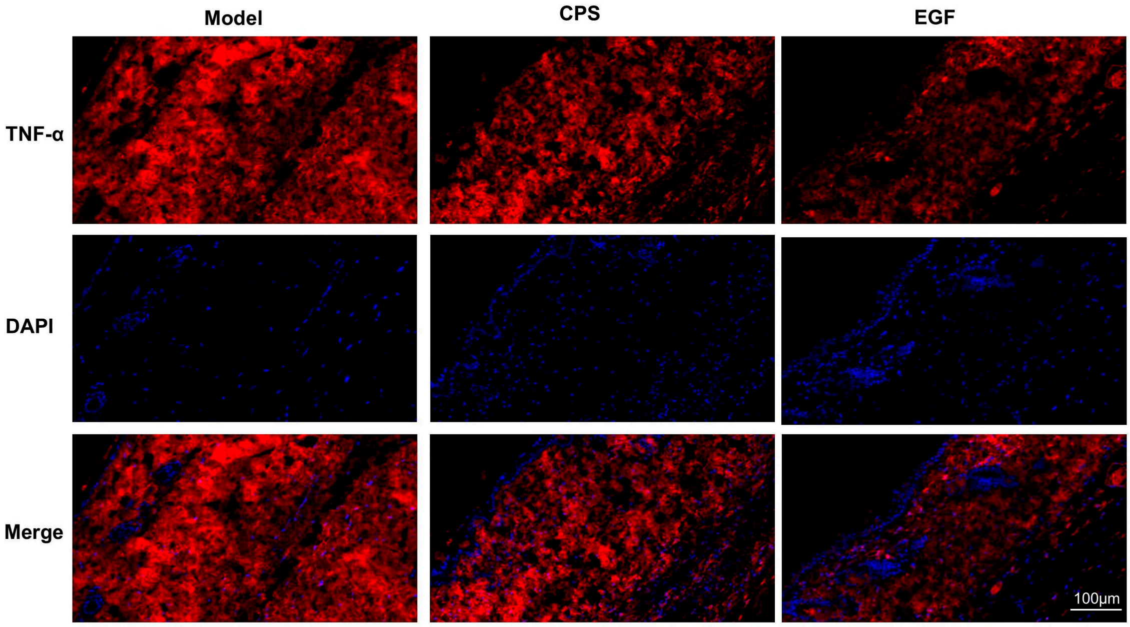

Figure S1.TNF-α fluorescence analysis

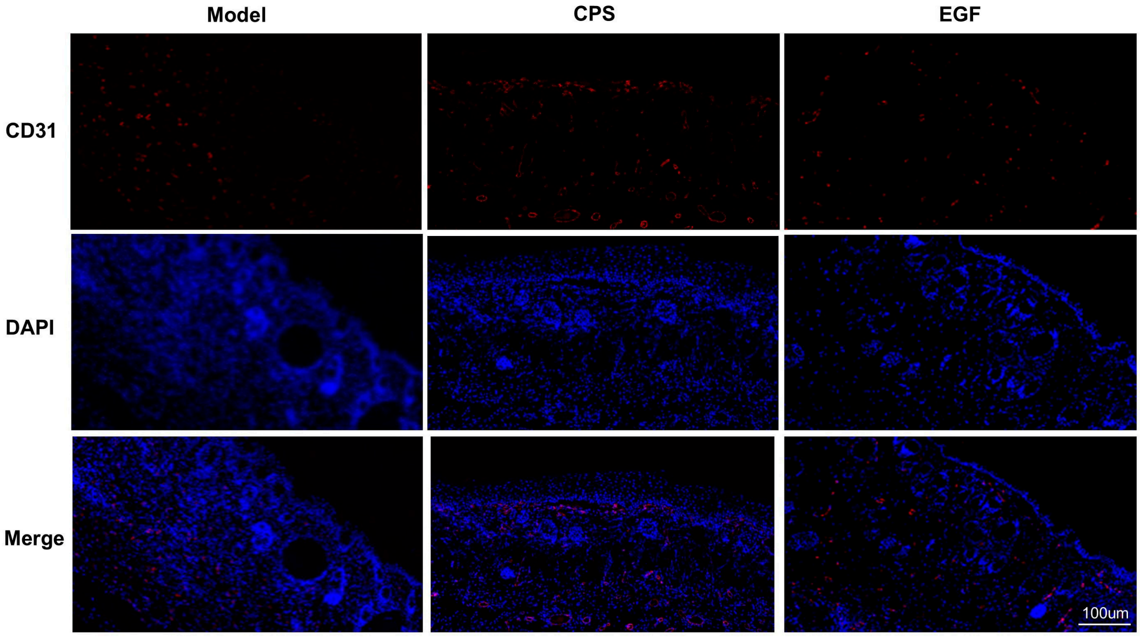

Figure S2.CD-31 fluorescence analysis on day 3

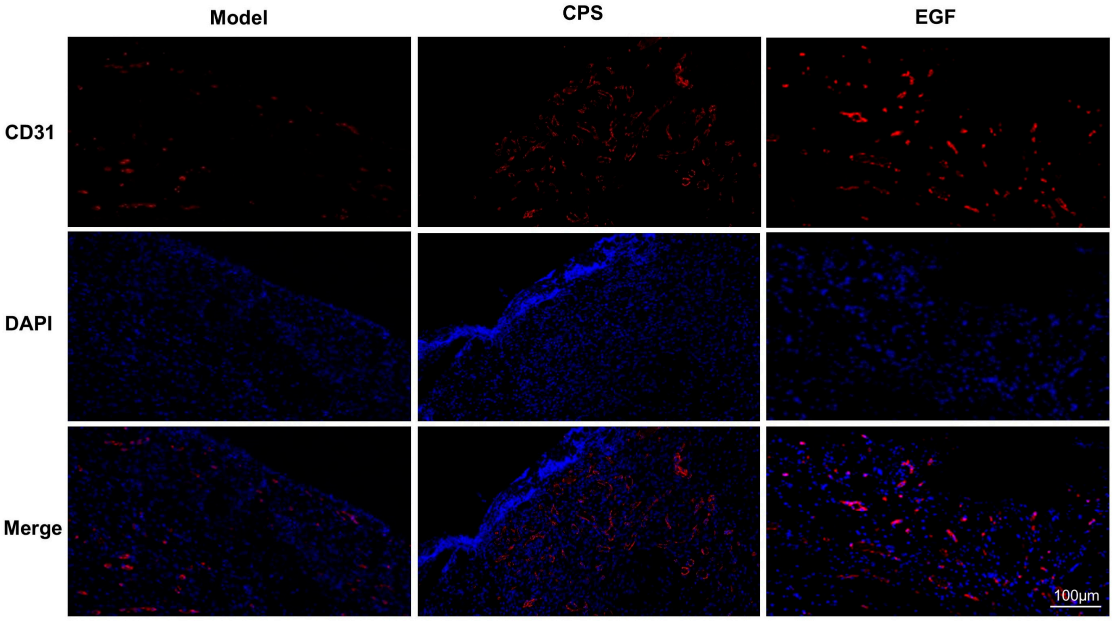

Figure S3.CD-31 fluorescence analysis on day 9

Characterization of CMP

CMP NMR

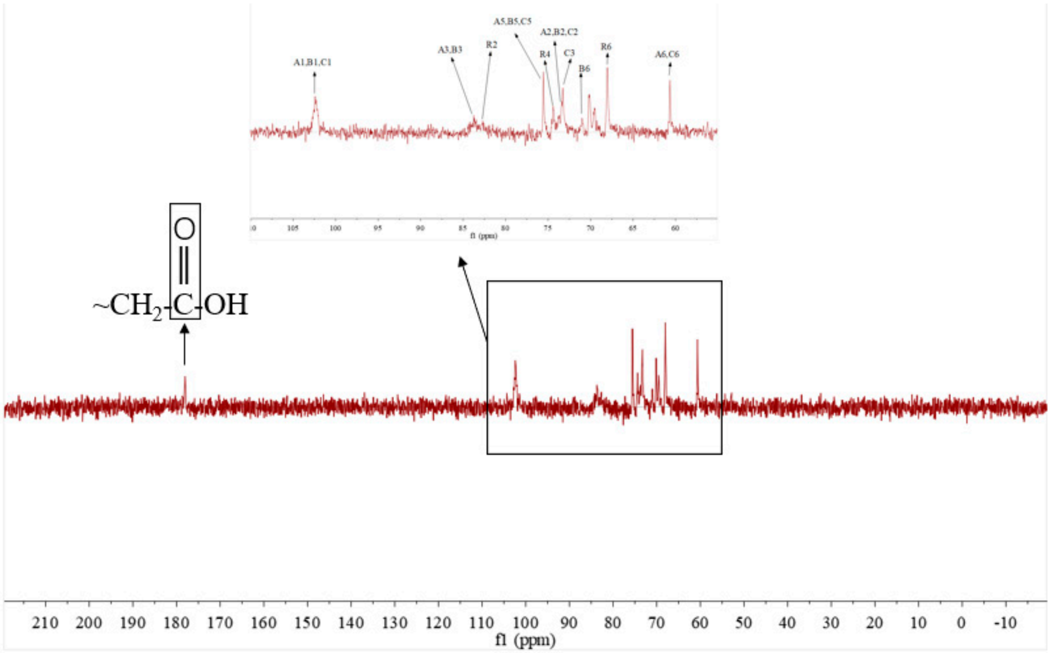

Figure S4. NMR of CMP

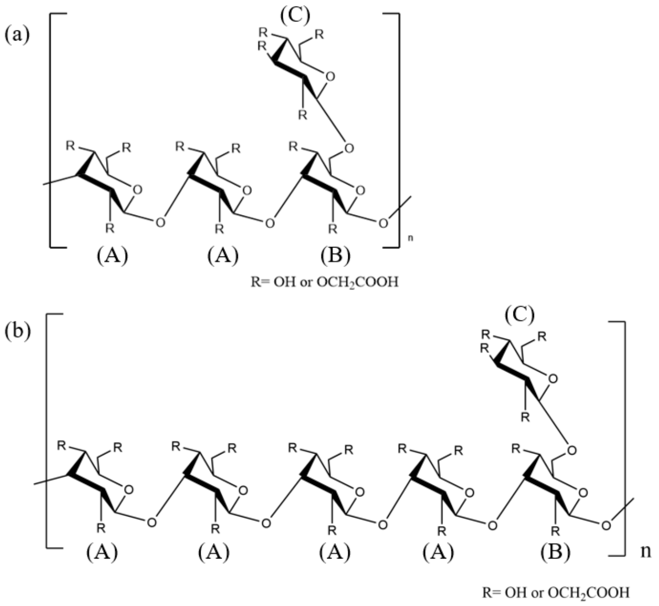

Figure S5. The structural formula of CMP

(a) Structure of carboxymethyl poria polysaccharide monomers

(b) Structure of long-chain carboxymethyl poria polysaccharides
